# Experimental Investigation on the Elicitation of Subjective Distributions

**DOI:** 10.3389/fpsyg.2019.00862

**Published:** 2019-04-23

**Authors:** Carlos J. Barrera-Causil, Juan Carlos Correa, Fernando Marmolejo-Ramos

**Affiliations:** ^1^Davinci Research Group, Faculty of Applied and Exact Sciences, Metropolitan Technological Institute, Medellín, Colombia; ^2^Escuela de Estadística, Universidad Nacional de Colombia, Medellín, Colombia; ^3^School of Psychology, The University of Adelaide, Adelaide, SA, Australia

**Keywords:** cluster analysis, expert knowledge elicitation, functional data analysis, prior distribution, subjective probability

## Abstract

Elicitation methods aim to build participants' distributions about a parameter of interest. In most elicitation studies this parameter is rarely known in advance and hinders an objective comparison between elicitation methods. In two experiments, participants were first presented with a fixed random sequence of images and numbers and subsequently their subjective distributions of percentages of one of those numbers was elicited. Importantly, the true percentage was set in advance. The first experiment tested whether receiving instructions as to the elicitation method would assist in estimating a true value more accurately than receiving no instructions and whether accuracy was determined by the numerical skills of the participants. The second experiment sought to compare the elicitation method used in the first experiment with a variation of a graphical elicitation method. The results indicate that (i) receiving instructions as to the elicitation method does assist in producing estimates closer to a true percentage value, (ii) the level of numerical skills does not play a part in the accuracy of the estimation (Experiment 1), and (iii) although the average estimates of the betting and graphical method are not significantly different, the betting method leads to more precise estimations than the graphical method (Experiment 2). Both studies featured statistical procedures (functional data analysis and a novel clustering technique) not considered in past research on the elicitation of subjective distributions. The implications of these results are discussed in relation to a recent key study.

## 1. Introduction

“The objective world is no more than a reflection of any person” (Tomás Carrasquilla, 1915)^1^.

When people are asked to provide numeric estimates of capital accumulations after a series of annual changes they tend to underestimate the accumulated financial growth even when they are to assume they have enough funds to cushion potential losses (Gonzalez and Svenson, 2014). People's responses thus rely on their subjective experience with and understanding of financial fluctuations and wealth. In other words, information about an uncertain parameter (e.g., an issue of interest) is essential for people to make decisions. Since this information relies on subjective experience acquired over time, it is thus conceivable that a person has various estimates, or proportion of estimates, for a specific parameter. This is a key component in Bayesian statistics known as the prior distribution (Berger, 1985).

In some instances, the only possibility is to work with an informative prior distribution, for example, in cases where sample data is unavailable, or the event will occur just once in a life. One illustration of this situation is the determination of the probability that an asteroid destroys the earth. In this case the researcher faces the need of eliciting an informative prior distribution based on personal knowledge (Schlag et al., 2015).

**Figure 1:**
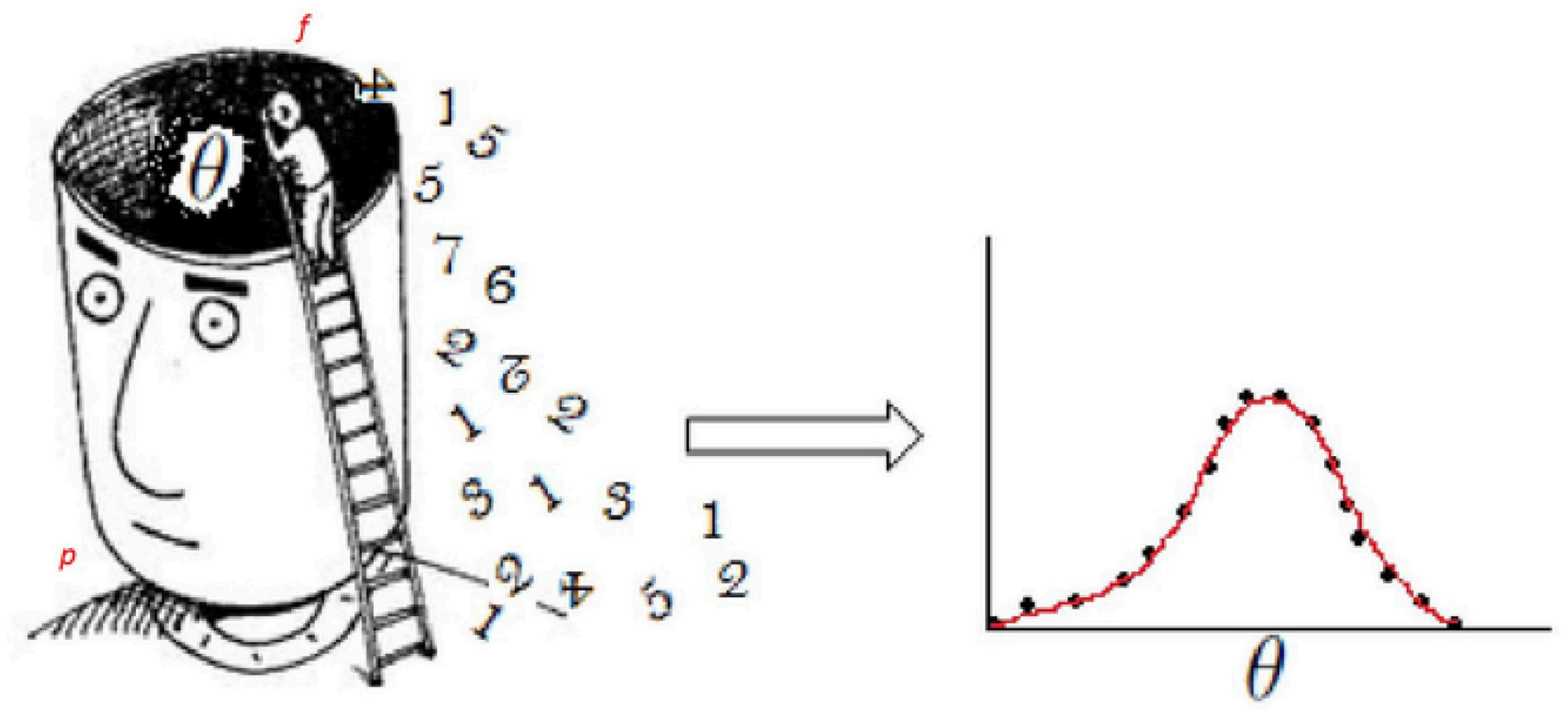
Illustration of the elicitation of priors. **Left**: a person (*p*) has knowledge-based experience that influences his/her beliefs about an issue of interest (θ). The dark area surrounding θ represents latent cognitive factors that also affect θ itself and the elicitation process. The person who elicits information about θ, or “facilitator” (*f*), has the task of reaching *p*'s beliefs. **Right**: as beliefs are largely qualitative, *f* also has to quantify them and render them into a probability distribution that captures what *p* knows about the issue (more technically, parameter) of interest θ. The distribution of θ can take any form in practice; for illustration purposes we showed a Gaussian distribution.

The elicitation of priors consists of extracting information about a parameter of interest from the subjective experience of a person and expressing it as a probability distribution (see Figure 1 and Anscombe and Aumann, 1963). So, if the elicitation process is applied to a group of persons, then the researcher will end up with several prior distributions. Indeed, several persons may have very different beliefs for the same parameter (Plous, 1993). However, different procedures are available to reduce several prior distributions to one. Winkler (1967, 1968, 1969) studied the problem of consensus in which persons produce several distributions that are combined into a single distribution to be used for posterior Bayesian analysis. For example, Albert et al. (2007) combined opinions from more than one person by using a hierarchical model that considers the bias and precision of the person as well as the consensus and diversity within the group. More recently, expert elicitation has been used in an educational context to foster teacher's self-reflection purposes (Lek and Van de Schoot, 2018).

Obtaining prior information is a very complex procedure that requires quantifying the knowledge of one or several participants in the area under study in order to build personal prior distributions (O'Hagan et al., 2006). Both the process of extracting information from the person's mind and the quantification of it are further affected by factors that increase the complexity of these procedures. Some of these factors are numerical skills and cognitive variables (Albert et al., 2007)^2^. For instance, attitudes have an effect in that they are context dependent (e.g., one's attitude differs when betting on a football game or picking a presidential candidate) (Plous, 1993). Research conducted by Hastorf and Cantril (1954) and Loy and Andrews (1981) are examples of this attitudinal changes. These individual characteristics thus suggest that the individual elicited prior distributions could represent different populations.

Due to individual differences (subjective experience), it cannot be guaranteed that different persons have the same grade of expertize or they have been exposed to the same events in their work. This is a default constraint that challenges the comparison of different elicitation techniques. An attempt to lessen this constraint was proposed by Wang et al. (2002) via an objective approach for evaluating an elicitation method that avoids the assumptions and pitfalls of existing approaches. However, their approach does not guarantee that people's knowledge is the same.

Traditionally, because elicitation methods have been compared in non-experimental situations (see Anscombe and Aumann, 2014), their results are not comparable. One reason for this is that people have different levels of knowledge and beliefs. Thus, if an elicitation method is applied to knowledgeable people (i.e., experts), it is very likely that their prior distributions will be good even if the elicitation method is deficient^3^. However, if the level of expertize of the persons is not controlled, it would be difficult to compare the elicitation methods. Also, this is impossible to achieve in real world situations.

One of the first comparisons of elicitation methods was proposed by Schweickert et al. (1987), where three techniques were used to extract the knowledge base from experts on lighting for industrial inspection tasks. Hudlicka (1996) compared three indirect knowledge elicitation techniques based on the number of attributes elicited, the ease with which these data were obtained, and the degree of post-analysis and interpretation required. In the same direction, Zhang (2007) compared three requirements elicitation techniques, but like in Schweickert and Hudlicka, this comparison did not control the level of the experts' knowledge^4^.

In this paper, we examine the resulting personal prior distributions about a percentage when participants receive or do not receive instructions about the elicitation process. Importantly, it is ensured that participants receive the same amount of information about a parameter of interest and a computer application is designed to elicit prior distributions via an interactive questionnaire. This interactive elicitation process provides a distribution of estimates for the parameter of interest for each participant. Further, a cluster analysis is carried out with the group who received elicitation instructions in order to detect if participants with different degrees of mathematical and/or statistical skills produce distributions of percentages that better capture the parameter of interest (Experiment 1). The elicitation method used in Experiment 1 is then compared with a variation of a graphical elicitation method (Experiment 2). Functional data analysis (FDA) techniques (see Wang et al., 2016) are used to characterize prior distributions of the participants and a novel method is used for clustering distributions (see Methods section for details) (Barrera and Correa, 2015).

## 2. Experiment 1

### 2.1. Participants

Fifty-nine undergraduate students verbally consented to volunteer for the experiment (age_range_ = 16–27). Of these participants, 14 had approved a course in basic mathematics and statistics at the university (mathematical and statistical skills group, G1; Mean_age_ = 21.7, *SD* = 2.8, females = 7), 26 had approved basic mathematics at the university (mathematical skills group, G2; Mean_age_ = 20.9, *SD* = 2.0, females = 11), and 19 had not completed either basic mathematics or statistics at the university (non-numerical skills group, G3; Mean_age_ = 22.8, *SD* = 2.6, females = 11). The study was carried out according to the Declaration of Helsinki (World Medical Association, 2013) and approved by the local ethics committee at the Metropolitan Technological Institute in Medellín-Colombia (ethical application ref: FGN-006).

### 2.2. Materials

The experiment was implemented in Microsoft Visual C++ and ran in a room hosting 40 computers with 2GHz Intel(R) Core(TM) i5–4590T processors and 8GB of RAM. Data were analyzed using R (R Development Core Team, 2016) using the add-on packages fda (Ramsay et al., 2014) and fda.usc (Febrero-Bande and Oviedo de la Fuente, 2012) for FDA, and cluster (Maechler et al., 2016) and clv (Nieweglowski, 2013) for cluster analysis.

### 2.3. Procedure

Participants were pseudo-randomly^5^ assigned into the elicitation instruction (I) and non-instruction (NI) groups (Table 1). Twenty-five participants formed the I group (Mean_age_ = 21, *SD* = 1.9, females = 12) and 34 participants formed the NI group (Mean_age_ = 22.2, *SD* = 2.8, females = 17).

**Table 1:** Random allocation of participants in the two experimental groups.

**Group**	**G1**	**G2**	**G3**	**Total**
Instruction (I)	6	13	6	25
Non-instruction (NI)	8	13	13	34
Total	14	26	19	59

*G1, mathematical and statistical skills; G2, mathematical skills only; and G3, non-numerical skills*.

Participants in the I and NI groups were informed they would see a random sequence of numbers and images and their task was to determine the percentage of times that the number one appeared (the actual value was 23% and each item was shown for 500 ms and with Interstimulus Interval; ISI = 0). In order to ensure both groups received the same input information, a fixed random order was used for the presentation of items (phase I). This part of the experiment lasted ~ 1 min. The random sequence of items consisted of 26 items; 10 ‘1' numbers, 10 ‘2' numbers, and six images.

Subsequently, both groups of participants underwent the elicitation process (phase II) but only those in the I group received instructions as to what the goal of the elicitation process was. The betting elicitation method was used. This is an interactive method in which the computer application asks questions and provides feedback to participants in order to gauge a range of minimum and maximum estimates and probability values for each. Specifically, the participant is asked about the bets he/she would be willing to place for or against the occurrence of a certain event (*E*). Assuming that *x*_*a*_ is the amount of money that a person is willing to bet for a total of *M* dollars, and that the utility function is linear, Cooke (1991) showed that the the expected utility of the betting is given by *MkP*(*E*) for some constant *k*, and that the expected utility of *x*_*a*_ is simply *kx*_*a*_. Setting these two expectations equal it follows that $P\left(E\right)=M^{-1}x_{a}$. In this work, we assume that utility functions are linear.

### 2.4. Statistical Analyzes

The goal of the elicitation process is to gauge data that can be used to build personal distributions for a specific parameter *θ* ∈ Θ, where Θ is the parameter space of *θ*.

Thus, let $A_{i}$ be fixed subintervals of Θ for the *i*-th participant (*i* = 1, 2, …, *n*) such that $A_{i}=\left[\theta_{1}^{i},\theta_{m}^{i}\right]$, with $\theta_{1}^{i}$ and $\theta_{m}^{i}$ correspond to the minimum and maximum value that *θ* can take according to the belief of the *i*-th participant, respectively. Now, let $A^{*}=\left[\theta_{1}^{*},\theta_{m}^{*}\right]={⋃}_{i=1}^{n}A_{i}$, and let us consider the grid $\theta_{1}^{*}<\theta_{2}^{*}<\cdots <\theta_{m-1}^{*}<\theta_{m}^{*}$. Thus, for the values ${\left\{\theta_{j}^{i}\right\}}_{j=1}^{m}$ of θ, each participant provides points (weights) ${\left\{y_{j}^{i}\right\}}_{j=1}^{m}$ which are represented in a graph; these points correspond to the levels of certainty that he/she has about each value in the sequence ${\left\{\theta_{j}^{i}\right\}}_{j=1}^{m}$. For example, if $\theta =\theta_{j}^{3}$, then $y_{j}^{3}$ would be the level of credibility that the third participant has about that statement.

For *n* participants, the above set up will result in a graph with *n* sequences of discrete and non-negative points ${\left\{\theta_{j}^{i},y_{j}^{i}\right\}}_{j=1}^{m}$ for *i* = 1, 2, …, *n*. FDA enables to represent the elicited priors in a continuous form by using numeric functions for curve fitting, such as *B*-splines, and to obtain information about measures that vary on a continuum (e.g., density curves and functional data like time-series). FDA makes use of descriptive measures, such as the functional mean, the (median) deepest curve, the functional boxplot, and analytical measures such as functional clustering methods. These measures are extensions of classical statistics methods, such as the mean, median, boxplot and the *k*-means clustering method (see Ramsay and Silverman, 2005 for technical details).

The cluster analysis is carried out here using a novel hierarchical clustering method, which works as follows. After obtaining the values ${\left\{\theta_{j}^{i}\right\}}_{j=1}^{m}$ and the corresponding certainty levels ${\left\{y_{j}^{i}\right\}}_{j=1}^{m}$ specified by the *i*-th participant (*i* = 1, 2, …, *n*), a *B*-spline is fitted to the ${\left\{y_{j}^{i}\right\}}_{j=1}^{m}$ of each participant. Doing so results in a grid of *k* points in the (0,1) interval, which corresponds to the range of possible values for the percentages of ones being displayed (in this study, *k* = 10,000). Further, a matrix of distances between these functions is obtained; this distance measure corresponds to the Hellinger's distance for the curves *x*^*s*^, *x*^*t*^ and is given by:

$$\begin{array}{l} d\left(x^{s},x^{t}\right)=\sqrt{\overset{k}{\underset{j=1}{\sum }}{\left(\sqrt{h_{j}^{s}}-\sqrt{h_{j}^{t}}\right)}^{2}}, \end{array}$$

where $h_{j}^{s}=\frac{y_{j}^{s}}{\sum y_{j}^{s}}$, $h_{j}^{t}=\frac{y_{j}^{t}}{\sum y_{j}^{t}}$, and $y_{j}^{s}$ and $y_{j}^{t}$ are the heights of the curves *x*^*s*^ and *x*^*t*^ in the point *j*, respectively.

Subsequently, the function hclust of R is used to construct a hierarchical cluster that uses this Hellinger's metric in combination with the Ward's method (see Murtagh and Legendre, 2014). This novel clustering method is used in this paper as a recent simulation study indicates this proposed method performs better than both agglomerative hierarchical clustering approaches, which combine Eucledian metrics with the unweighted pair-group arithmetic average method, and the Ward's method (Barrera and Correa, 2015).

Location and scale estimations are reported via the Mean and the standard deviation (SD) and bias-corrected-and-accelerated (BCA) (Efron, 1987) confidence intervals (CI) via bootstrap are estimated for values of interest.

#### 2.4.1. Hellinger Distance

We know that Euclidian distance is sensitive to the measurement units of the variables. Therefore, changes in scale affect changes in the distance between individuals. In this paper, we use prior distributions with different symmetries and kurtoses. Thus, changes in the heights of the curves, may represent problems in the Euclidean metric. In scenarios like this the Hellinger distance is more appropriate for density functions and adaptable to discrete distributions (Cuadras and Fortiana, 1993).

There are ways to measure distances between probability measures and these distances do not depend on the parametrizations. In probability and statistics, the Hellinger distance is used to quantify the similarity between two probability distributions without depending on the parametrizations (van der Vaart, 2000).

The Hellinger distance between two probability measures is the *L*_2_-distance between the square roots of the corresponding densities in terms of the elementary probability theory. If we denote the densities as *f* and *g*, respectively, the squared Hellinger distance can be expressed as a standard calculus integral (van der Vaart, 2000)

$$\begin{array}{l} \int {\left(\sqrt{f\left(\theta \right)}-\sqrt{g\left(\theta \right)}\right)}^{2}d\theta . \end{array}$$

For two discrete probability distributions *P* = (*p*_1_…*p*_*m*_) and *Q* = (*q*_1_…*q*_*m*_), their Hellinger distance is defined as

$$\begin{array}{l} H\left(P,Q\right)=\sqrt{\overset{m}{\underset{i=1}{\sum }}{\left(\sqrt{p_{i}}-\sqrt{q_{i}}\right)}^{2}}. \end{array}$$

### 2.5. Results

**Figure 2:**
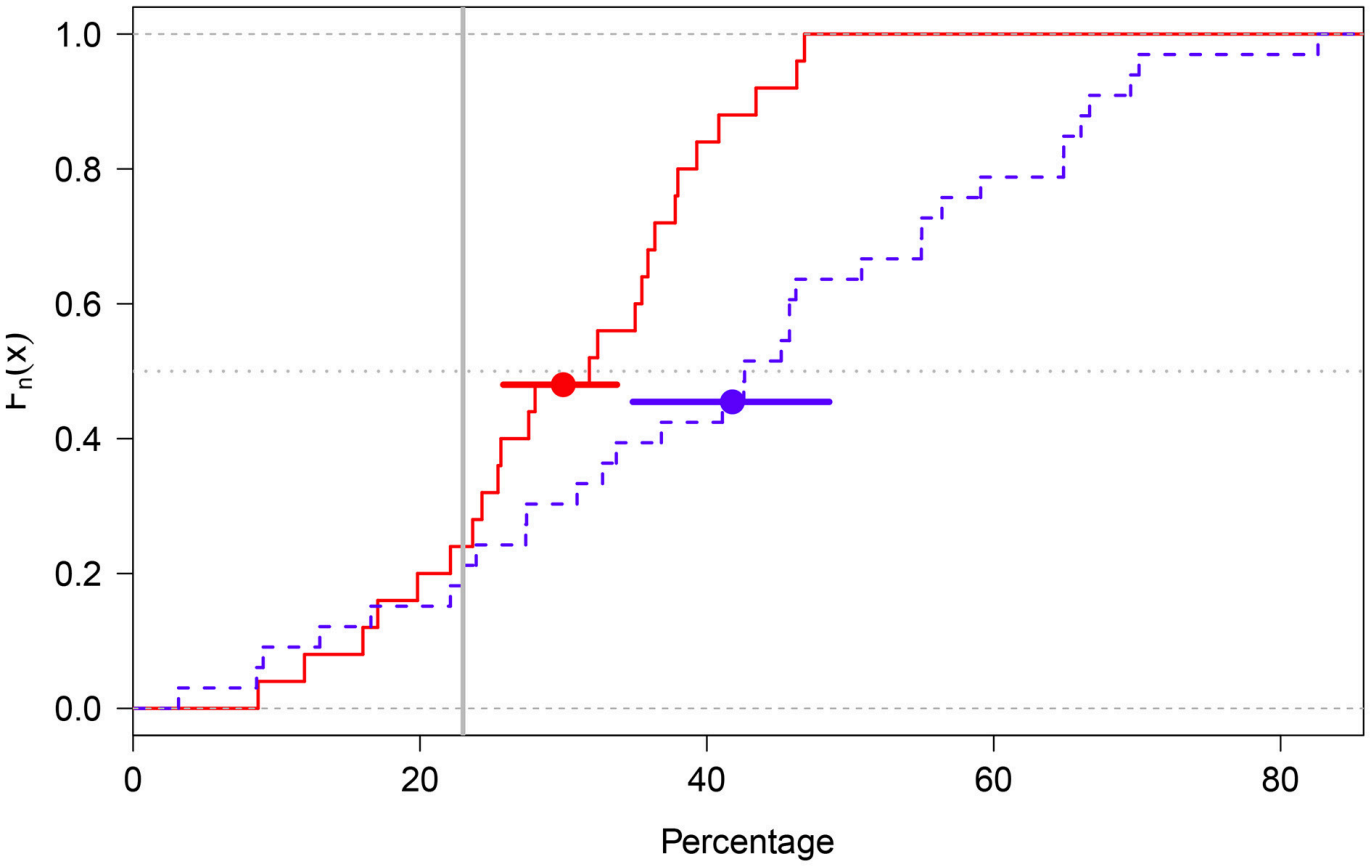
Empirical cumulative distribution function plot of the I (red solid line) and NI (blue dotted line) groups. The groups' means are shown as solid dots. The error bars around the means represent 95%_BCA_CIs. The gray dotted horizontal line cuts across the groups' median values and the gray solid vertical line represents the true percentage value (23%).

A test of the difference between the average values of the I and the NI groups was carried out by calculating the median value in each participant's distribution of percentages, and then performing a Welch *t*-test comparing the means of the two resulting distributions. The parametric pairwise comparison was performed via Q–Q plots (Vélez and Correa, 2015; Loy et al., 2016) and the Shapiro-Wilk (SW) normality test (Marmolejo-Ramos and González-Burgos, 2013), indicating that data in the I and NI groups are normally distributed (*p*_SW = 0.70 in both groups). The pairwise comparison also indicated the average percentages of ones in the I and NI groups (I group: Mean = 29.98%, 95%_BCA_CI = [25.79,33.83]; NI group: Mean = 41.77%; 95%_BCA_CI = [34.62,48.57]) were statistically significantly different (*t*_49.94_ = −2.85, *p* = 0.006; see Figure 2)^6^.

**Figure 3:**
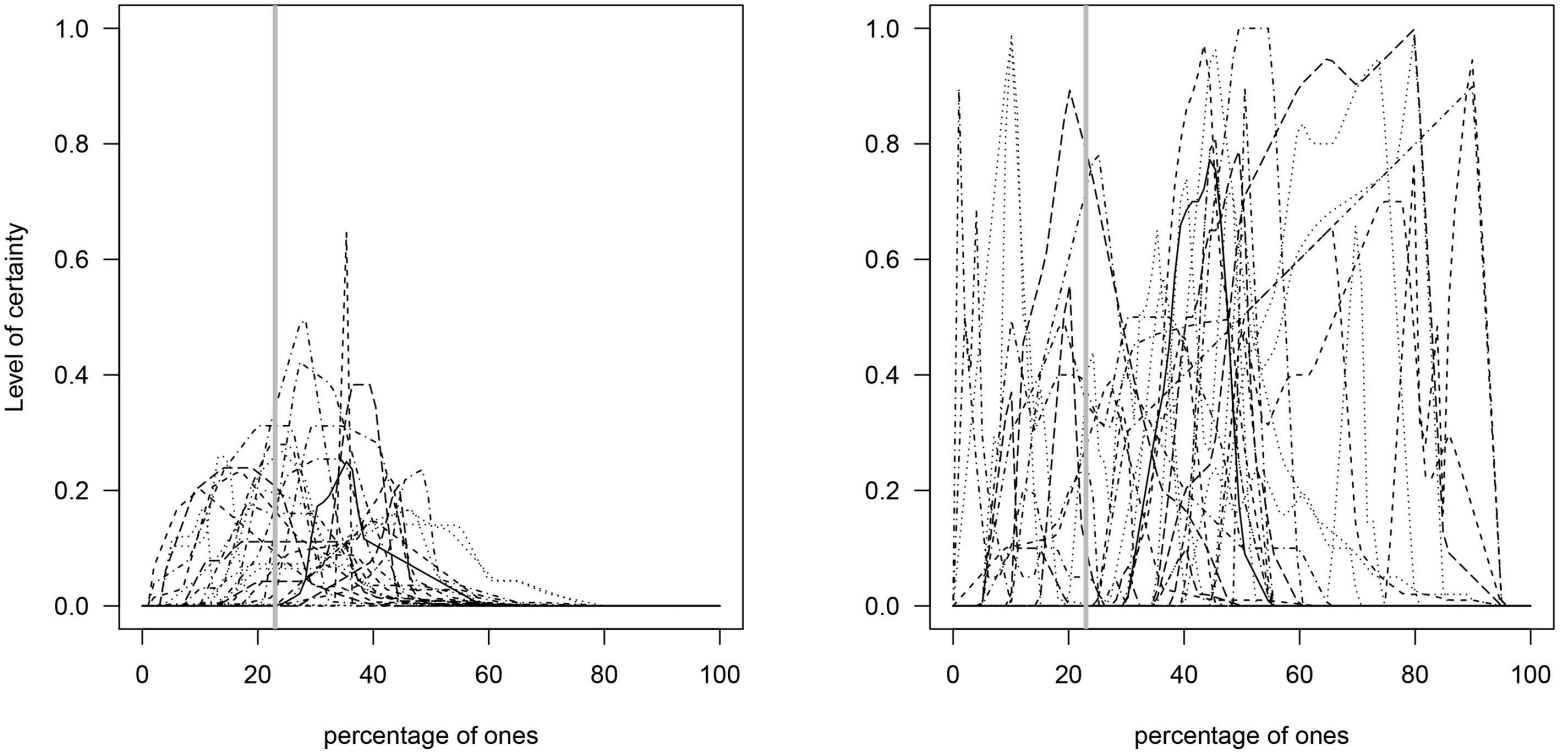
Elicited prior distributions of the percentages given by each of the participants in the I **(Left)** and NI **(Right)** groups. The gray solid vertical line represents the true percentage value (23%).

**Figure 4:**
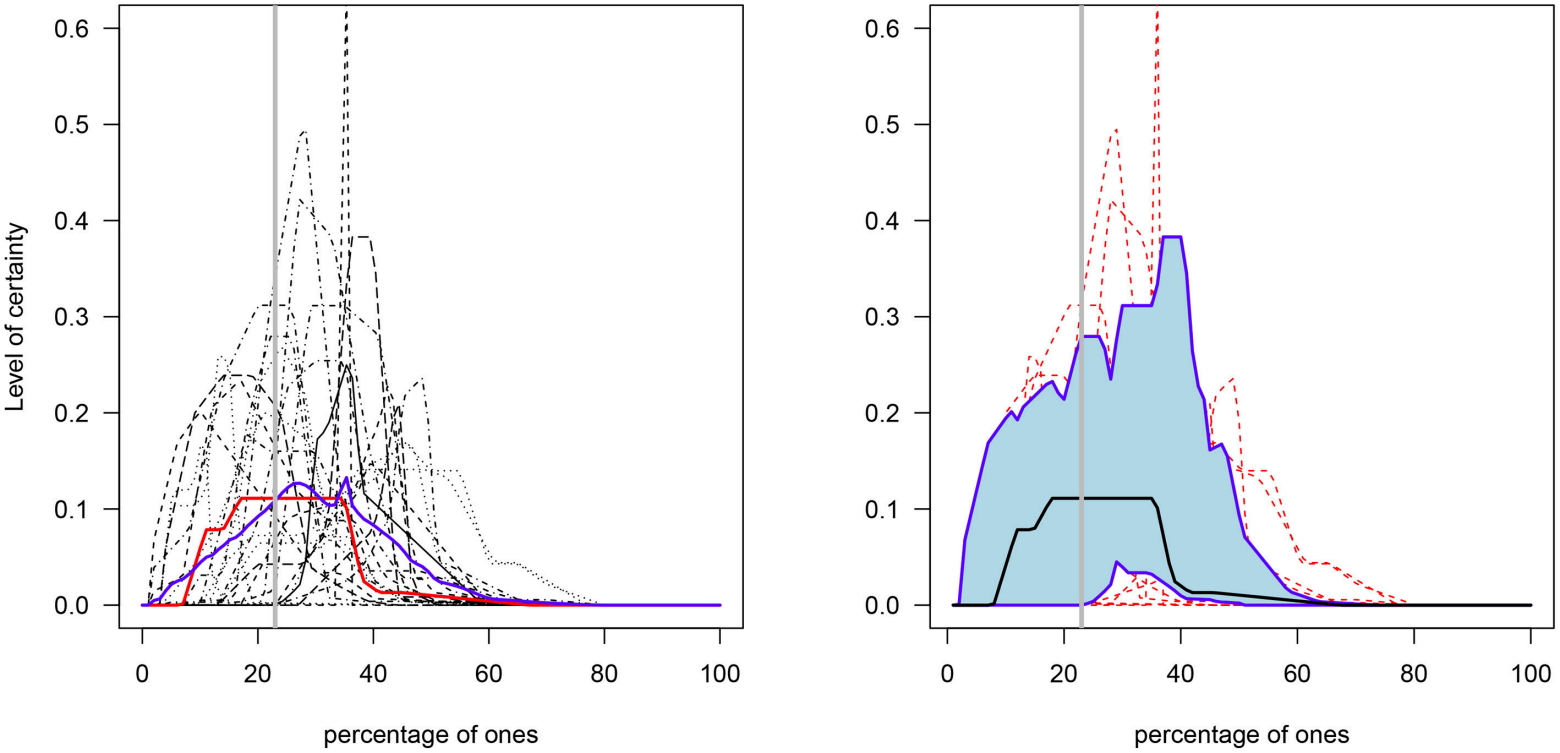
Elicited prior distributions of the percentages in the I group. **Left**: the blue and red lines represent the functional mean and median curves, respectively. **Right**: the solid black line represents the median curve and the lower and upper blue solid lines represent the functional first and third quantiles, respectively (individual distributions are shown in the background in red dotted lines). The gray solid vertical line represents the true percentage value (23%).

These results thus suggest that participants in the NI group had more difficulties than participants in the I group in estimating percentages close to the true value (23%). In other words, explaining what the elicitation process was about (i.e., its goals and steps) assisted participants in the I group to produce estimates closer to the true value (see Figure 3). Indeed, a closer look at the distributions obtained in the I group indicates their median deepest curve has narrower spread around the true value than their mean curve (median curve = 25.3% and mean curve = 29.3%) (Figure 4).

**Figure 5:**
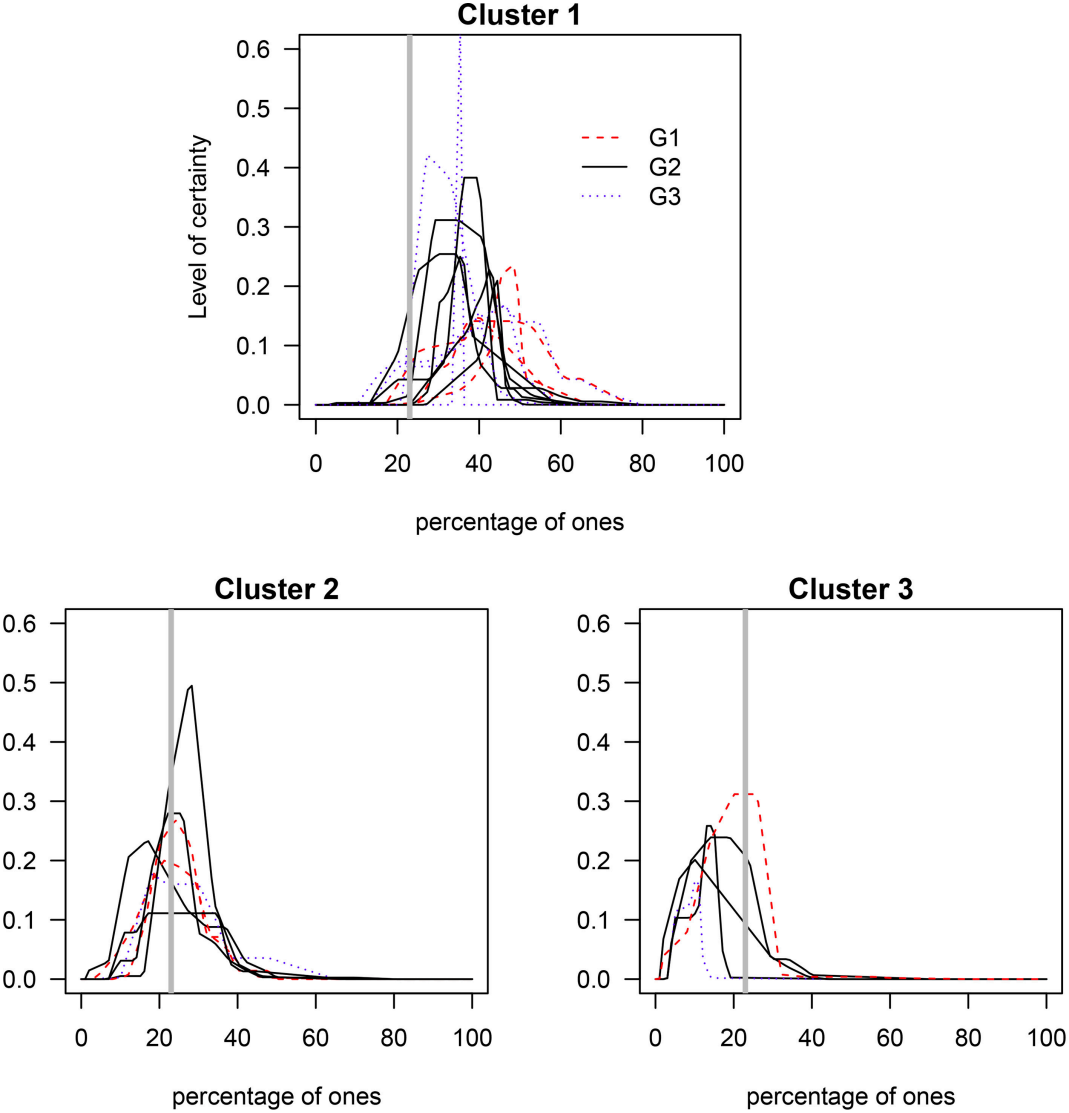
Clusters of the elicited prior distributions of the three groups with varying mathematical and/or statistical skills. G1, mathematical and statistical skills group; G2, mathematical skills group; and G3, non-numerical skills group. The gray solid vertical line represents the true percentage value (23%).

**Figure 6:**
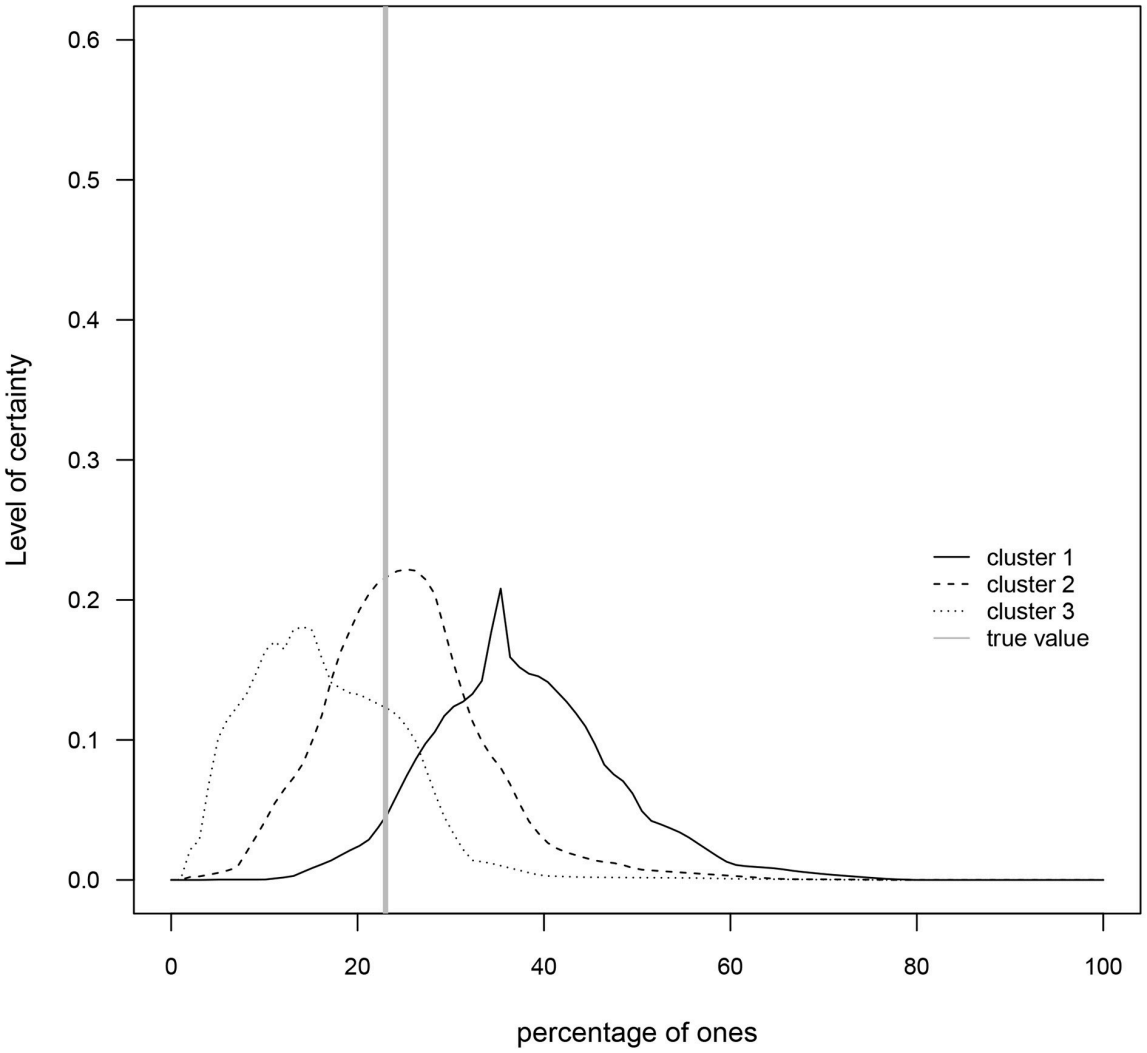
Prior means for each cluster. The gray solid vertical line represents the true value of the percentage of ones (23%).

A cluster analysis was performed on the I group data in order to investigate if members of the G1, G2, and G3 groups (Table 1) generated distributions for the percentage of ones that better capture the true value^7^. That is, the goal is to determine whether the three levels of numerical skills are reflected in clusters of skills such that those with the highest level exhibit distributions closer to the true value. The results indicate that around 50% of participants in each of the three groups were grouped in cluster 1, around 33% were grouped in cluster 2, and ~ 17% were grouped in cluster 3 (see Table 2). As Figures 5, 6 show, cluster 2 grouped those participants whose distributions' highest levels of certainty were closer to the true value. In clusters 1 and 3 the true value occurred, respectively, on the lower and upper areas of the distributions' tails.

**Table 2:** Clusters of the three groups with varying mathematical and/or statistical skills.

**Cluster**	**G1**	**G2**	**G3**	**Total**
1	3	6	4	13
2	2	4	1	7
3	1	3	1	5
Total	6	13	6	25

*G1, mathematical and statistical skills group; G2, mathematical skills group; and G3, non-numerical skills group*.

These results thus indicate that the level of numerical skills do not determinate the confirmation of clusters. That is, the clusters were conformed by a mixture of participants representing three levels of numerical skills and the cluster that better captured the true value was indeed no different in this regard. Although unknown cognitive factors (e.g., fatigue) and other demographics (e.g., gender) could have had an effect on the prior distributions obtained for each participant, it is also likely that the method used to build such distributions has had an effect. The elicitation method itself is therefore central to the construction of personal prior distributions about a parameter of interest. This experiment showed that the betting (elicitation) method did help participants to build their prior distributions but it is open to question if another elicitation method could have led to a comparable outcome. Experiment 2 had thus the goal of comparing the betting method with a method that elicits knowledge via probability distribution plots.

## 3. Experiment 2

### 3.1. Participants

Thirty-three undergraduate students verbally consented to volunteer in the experiment (Mean_age_ = 21.9, age_range_ = 17–29, *SD* = 2.5, females = 16). None of the participants was involved in Experiment 1. The study was carried out according to the Declaration of Helsinki (World Medical Association, 2013) and approved by the local ethics committee at the Metropolitan Technological Institute in Medellín-Colombia (ethical application ref: FGN-006).

### 3.2. Materials

As in Experiment 1.

### 3.3. Procedure

**Figure 7:**
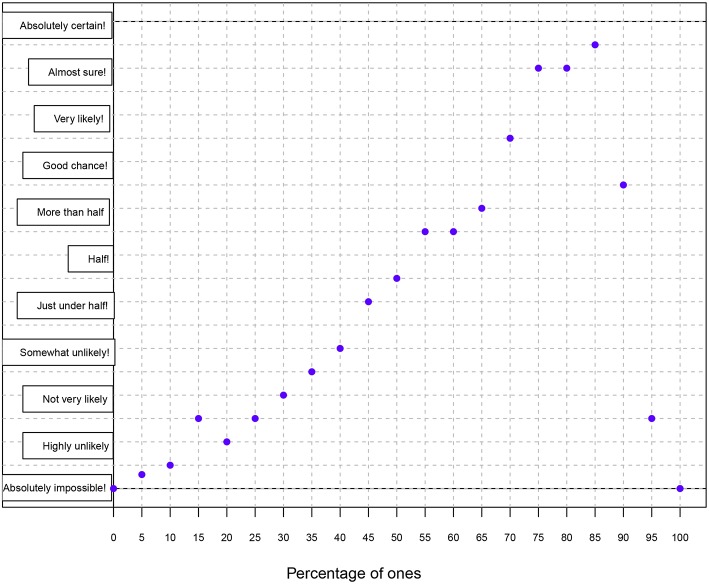
Illustration of the graphical elicitation method. The participant sees a grid without dots and his/her task is to assign a degree of probability (*Y* axis) to each of the percentage values (*X* axis). The *Y* axis represents degrees in probability via 11 linguistic forms (from bottom to top: absolutely impossible, highly unlikely, not very likely, somewhat unlikely, just under half, half, more than half, good chance!, very likely!, almost sure!, and absolutely certain!) (Mosteller and Youtz, 1990).

Participants were randomly assigned into two groups: the betting (B) and graphical (G) elicitation groups. The betting elicitation method was the same used in Experiment 1, with the consideration that people were instructed before the elicitation session. The graphical elicitation method enables to represent the degree of knowledge about a parameter of interest via histograms, smooth curves (akin to probability density function plots), or points in the Cartesian plane (Chesley, 1975). The ultimate goal is therefore to approximate a probability distribution. In this method, participants are asked to pinpoint on a grid of possible values the level of certainty they have about a parameter. While the *X* axis represents the values the parameter of interest can obtain, the *Y* axis represents degrees in probability via adjectives or adverbs of frequency (see Mosteller and Youtz, 1990; Renooij and Witteman, 1990) (Figure 7).

Fifteen participants formed the B group (Mean_age_ = 21.5, age_range_ = 17–24, *SD* = 2, females = 8) and 18 participants formed the G group (Mean_age_ = 22.3, age_range_ = 19–29, *SD* = 2.9, females = 8). As in Experiment 1, participants in both groups were informed they would see a random sequence of numbers and images and their task was to determine the percentage of times that the number one appeared (the actual value was 77% and each item was shown for 500 ms with Interstimulus Interval ISI = 0). In order to ensure both groups received the same input information, a fixed random order was used for the presentation of items (phase I). This part of the experiment lasted ~ 1 min. The random sequence of items was the same used in Experiment 1. Subsequently, both groups of participants underwent the elicitation process (phase II).

### 3.4. Statistical Analyzes

As in Experiment 1, FDA tools were used.

### 3.5. Results

**Figure 8:**
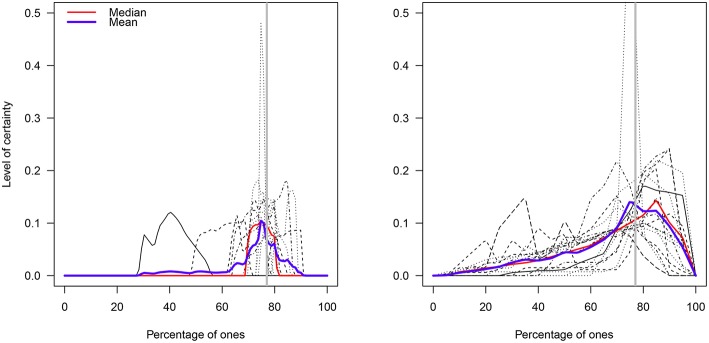
Prior distributions of the percentage of ones in the B and G elicitation groups. The blue and red lines represent the functional mean and median curves, respectively. The gray solid vertical line represents the true percentage value (77%).

**Figure 9:**
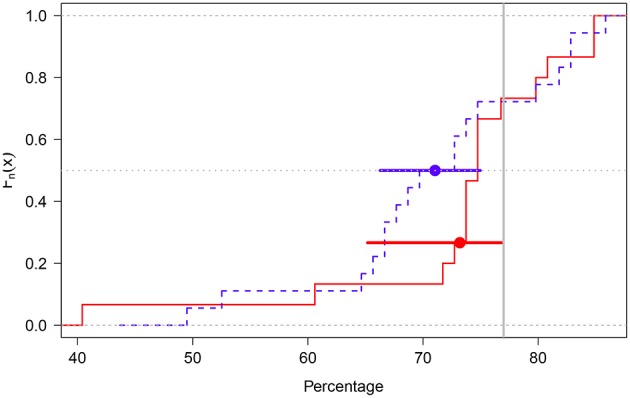
Empirical cumulative distribution function plot of the B (red solid line) and G groups (blue dotted line). The groups' means are shown as solid dots. The error bars around the means represent 95% BCA confidence intervals. The gray dotted horizontal line cuts across the groups' median values and the gray solid vertical line represents the true percentage value (77%).

The individual distributions for each elicitation method are shown in Figure 8. As in Experiment 1, the median value in each participant's distribution of percentages was estimated and the two resulting distributions were compared via a Welch *t*-test. This test suggested the groups' mean percentages (B group: M = 73.19%; 95%_BCA_CI = [65.18,76.83]; G group: M = 71.04%; 95%_BCA_CI = [66.27,74.97]) did not statistically differ (*t*_28.77_ = 0.59, *p* = 0.55; Figure 9)^8^.

A visual analysis suggested that although the B group was more left-skewed than the G group (due to two very low median values: 40.4 vs. 60.6%; Figure 9), the B group had less variability than the G group (MAD_B_ = 2.99; MAD_G_ = 7.48). Indeed, when the two outlying values were removed from the data in the B group (the prior distribution of the participants were illogical respect to their values), this group exhibited average percentages that included the true value (M = 76.68%; 95%_BCA_CI = [74.66,79.40]).

## 4. Discussion

The first study set out to investigate if receiving instructions as to the elicitation method would assist in estimating a true value more accurately than receiving no instructions and whether accuracy was determined by the numerical skills of the participants. The second study sought to compare the elicitation method used in Experiment 1 with a variation of a graphical elicitation method. As to the Experiment 1, the results suggest that receiving instructions as to the elicitation method does assist in producing estimates closer to a true percentage value and the level of numerical skills does not play a part in the accuracy of the estimation. In regard to Experiment 2, the data indicate that although the average estimates of the betting and graphical method are not significantly different, the betting method leads to more precise estimations than the graphical method. Methodologically speaking, both studies featured statistical procedures (FDA tools and a novel clustering technique) not considered in past research on the elicitation of subjective distributions. The implications of these results are discussed in relation to a recent key study.

Grigore et al. (2016) compared the histogram (graphical) and the hybrid elicitation methods in order to obtain subjective probability distributions as to the cost-effectiveness analysis of alternative treatments for prostate cancer. Their results showed that although participants gave more positive ratings to the graphical than to the hybrid method^9^ as to the ease of use, the hybrid method was assessed as more accurate. If we entertain the idea that the hybrid method is somewhat akin to the betting method, the results of our Experiment 2 indicate that non-graphical methods seem to lead to estimates closer to the true value (see Figure 9).

According to the results of Grigore et al. (2016), the graphical method exhibited less variability around the location parameter than the hybrid method. These results differ from what our Experiment 2 showed in that the graphical method had more variance than the betting method. Interestingly, though, Grigore et al. (2016) found that the location parameters obtained via the graphical method were lower than those given by the hybrid method. Our Experiment 2 also showed that the graphical method lead to lower average estimations of the true parameter than those given by the betting method. Thus, although the graphical method seems easy to use, other methods (e.g., the betting and the hybrid methods) tend to shift participants distributions toward more precise estimates. Having said this, graphical methods need to be tested under different scenarios in order to assess their usability. For example, one could speculate that graphical methods could lead to more homogeneous distributions and accurate estimates than other elicitation methods when the parameter of interest refers to a topic relevant to participants who quotidianly rely on graphical displays (e.g., graphic designers, architects, or researchers on statistical graphics). Indeed, research on the assessment of normality of data distributions indicates that graphical displays can be more powerful than traditional goodness of fit tests (see Loy et al., 2016). The key message therefore is that rich information can be extracted from a simple visual assessment of probability distributions. Thus, the elicitation of subjective probabilities via graphical displays demands further investigation.

In Experiment 1, we found that walking the participant through the elicitation method does help in building subjective distributions with low variance around an average estimate that is close to the true value compared to not doing so (see Figure 2). Our elicitation sessions (I group in Experiment 1, and B and G groups in Experiment 2) resembled that used by Grigore et al. (2016) (see section “elicitation sessions” in their article). However, a key extra step performed by these authors was to have the participants provide ratings as to the ease of completion of the elicitation method, their face validity, and comments (via open questions) as to the task itself. We did not include such extra questions but we believe it is something to be aware of for future elicitation experiments. In our Experiment 1, though, we assessed participants numerical skills since this was a variable of explicit interest in our study and, as the results indicated, it seems to have no effect on the precision of the true estimate. Nevertheless, we believe that extra information as to the participants (e.g., basic demographics and emotional and cognitive states) needs to be used for weighting their distributions. FDA tools can be used to build subjective distributions and the cluster method proposed in Experiment 1 can be used to re-group subjective distributions according to variables of interest. We believe using these statistical tools in the context of the elicitation of priors enables to build more accurate subjective distributions and perform proper distributional analyzes^10^.

A point that we believe needs extra attention and is a central step in familiarizing the participant with the elicitation process is to explain to participants general concepts in probability. Recent brain imaging evidence suggests that while assessing prior probabilities (i.e., the degree of prior certainty) requires frontal brain activation, assessing likelihoods correlates with parietal activation (Kopp et al., 2016). It might be the case that the definitions given to participants as to what probability entails could reflect not only on their brain activations but also on their statistical behavior. In most research of elicitation, probability seems to be understood as a blend between frequency distributions and hypotheses (e.g., opinions) for measuring relative degrees of uncertainty (Monari, 2015). However, probability has also been defined as a pure mathematical concept and as propensity (natural tendency of a concrete thing to be in a certain state or to experience certain changes) (Bunge, 1981). These definitional issues need to be stated and clarified in elicitation studies.

## Ethics Statement

All participants received course credit or participated voluntarily. The study's protocol was approved by the ethics committee of the Instituto Tecnologico Metropolitano. All subjects gave written informed consent in accordance with the Declaration of Helsinki.

## Author Contributions

CB-C and JC conceived and design of the study. CB-C organized the database. CB-C and FM-R performed the statistical analysis. CB-C wrote the first draft of the manuscript. JC and FM-R wrote sections of the manuscript. All authors contributed to manuscript revision, read, and approved the submitted version.

### Conflict of Interest Statement

The authors declare that the research was conducted in the absence of any commercial or financial relationships that could be construed as a potential conflict of interest.

## Footnotes

^1^This phrase appears in a short essay titled “Elogio de la viuda sabia” (In praise of the wise widow) published in a Colombian newspaper and for which, to our knowledge, there is no published English translation. The original sentence reads as: “El mundo objetivo no es más que un reflejo de cualquier sujeto.”

^2^Some of those cognitive biases and variables are representativeness, availability, adjustment, and anchoring (Kahneman and Tversky, 1972; Tversky and Kahneman, 1974), range-frequency, and overconfidence (O'Hagan, 2019). These cognitive variables play a key role in expert knowledge elicitation and thus need further investigation.

^3^A prior distribution could be considered as good enough when it correctly reflects the expert's previous beliefs and do not suggest quite different plausible regions for the parameters of interest.

^4^Other techniques, such as interviewing, protocol analysis, multidimensional scaling, logistic regression (Wright and Ayton, 1987), and item response theory (Andrade and Gosling, 2018) have also been proposed.

^5^Initially, participants were randomly assigned into two balanced groups (i.e., 30,30), but a failure in one of the computer rooms and the absence of one participant led to having two unbalanced groups.

^6^These statistics and the ECDFs were estimated after one outlying observation in the NI group was removed. Such outlier (value = −4.66) was the median percentage of a participant's distribution who exhibited very low and illogical values and the *B*-spline smoothing simply exacerbated such result. A pairwise comparison between the I and NI groups remained significant even when such outlier was not excluded (*t*_50.27_ = −2.44, *p* = 0.018).

^7^A permutation test of the equality of two density estimates (Bowman and Azzalini, 2014) indicated the distributions of functional means were different (the FDR-adjusted *p*-values of the three comparisons were close to zero).

^8^Because the Shapiro-Wilk normality test indicated that the data in the group B did not distribute normally (*p* < 0.001), groups were also compared using the Wilcox rank sum test with continuity correction. This test also showed no difference in the mean percentage of ones (*W* = 167.5, *p* = 0.24).

^9^In this method, participants are first prompted to give the highest and lowest possible estimates for the parameter of interest; then, intervals within the highest and lowest values are built and the participant is required to assign probabilities to each interval.

^10^The topic of distributional analysis is essential to research on the elicitation of distributions. Other tools that are designed to deal with distributions and that should be considered in future studies are Generalized Additive Models for Location Scale and Shape (GAMLSS) (Stasinopoulos and Rigby, 2007), Linear Quantile Mixed Models (LQMM) (Geraci and Bottai, 2014), and finite mixture distributions (McLachlan and Peel, 2000).
